# Items of General Interest

**Published:** 1915-03

**Authors:** 


### Items of General Interest.

During the month of February eleven cases of smallpox were under quarantine in Wise county.

It is announced by Drs. Seymour Oppenheimer and Mark J. Gottlieb of New York that there is a cure for hay fever.

Dr. R. E. Owens of Dalhart has established a branch hospital in connection with Guyton Nichols' sanitarium at Plainview.

The Moose have decided to build a sanitarium for the cure and treatment of members of the order who may be suffering with tuberculosis, and it is thought that El Paso will get the sanitarium.

It is announced by Dr. Holman Taylor that the attendance of the Texas State Medical Association, which will meet in Fort Worth in May, will be the largest in the history of the organization.

Charges of practicing medicine without licenses were made on informations filed in the county criminal court of Fort Worth against William Hager, Mrs. E. E. Hargett, Gordon Hargett, G. Haggard Rider, and C. E. Hyroop.

Representative Williford of the Texas Legislature has introduced a bill (by request) to give practicing physicians a lien on their patrons' crops, the lien to come before any other lien save that of the landlord and the laborer.

Drs. William and Charles Mayo of Rochester, world famous surgeons, propose to establish a $1,000,000 foundation for medical research and to place the foundation under certain restrictions in the hands of the University of Minnesota Board of Regents.

One of this JOURNAL's contributing editors, Dr. Walter S. Sutton of Kansas City, sailed for Europe February 15 to take service in a hospital established by Mrs. Whitney. We shall hope for some interesting sketches from Dr. Sutton.

The next meeting of the South Texas District Medical Association will be held at Victoria April 8 and 9.

The following announcement of the sections with the names of chairman and secretary assure the success of the meeting:

Section on Surgery: Chairman, Dr. J. H. Reuss, Cuero; secretary, Dr. J. M. O'Farrell, Richmond.

Section on Obstetrics and Gynecology: Chairman, Dr. Geo. H. Lee, Galveston; secretary, Dr. Karl Chambers, Jasper.

Section on Public Health: Chairman, Dr. C. C. Green, Houston; secretary, Dr. B. L. Arms, Galveston.

Section on Medicine and Diseases of Children: Chairman, Dr. Will P. Coyle, Orange; secretary, Dr. S. A. Foote, Bay City.

Dr. W. F. Thomas of Beaumont, the secretary of the association, urges a large attendance, and cleverly says, alliteratively, "Let's Visit Vigorous Victoria with a Vim."

The many friends of the Lambert Pharmacal Company will be pleased to know that they have won their infringement suit against the Bolton Chemical Co., who are manufacturers of a preparation known as Listogen. The suit was decided on January 11, 1915.

The Medical Society of the State of New York announces that it will hold its hundred and ninth annual meeting in Buffalo April 27-29. On account of the European war this will probably be the largest medical meeting of the year, except perhaps that of the A. M. A. in San Francisco. Through the co-operation of the military authorities the meeting will be held in the Sixty-fifth Regiment Armory—not the old arsenal, now the City Convention Hall. This armory is one of the largest in the country and will afford accommodations for all activities of the meeting, except the annual banquet. A restaurant will be conducted in the building; there will be ample space for commercial and scientific exhibits, and an abundance of halls for general and section meetings. Even an automobile park will be provided on the armory grounds. No one need leave the building except to sleep, unless possibly to attend lectures to the laity which will be given by prominent visiting physicians and which will probably be held in the Masten Park High School across the street. The choice of the armory is fortunate in another sense, as indicating the organization of the State Society as an arm of the State government. On the last night of the meeting a regimental parade and review by General Gorgas will be held. All physicians are cordially invited to attend.

SPECIAL MAIL COURSE FOR HEALTH OFFICERS.

A correspondence course for health officers is announced by the University of Wisconsin extension division. This course has been prepared to meet the need and desire frequently expressed for better preparation for local health administration. It is designed for health officers not able to pursue or warranted in taking residence work, as well as for others desiring to take up the study of health administration.

But few communities are prepared to employ on full time thoroughly trained sanitarians, according to the bulletin announcing the courses. Yet there is not a single community that does not need expert sanitary work in some direction. The topics treated in this course will cover laws and regulations, vital statistics and health surveys, transmission of disease, nuisances, and administration of a health department. The administration part of the course will treat of inspection work, visiting nursing, medical inspection of school children, quarantining, isolation, and disinfection, use of laboratory, registration and other subjects.

---

It is claimed by Dr. J. H. Wilms of Cincinnati that he has discovered an antidote for bichloride of mercury poisoning.

Dr. H. Bergman, county health officer of Polk county, reports about forty cases of smallpox in the upper end of that county.

---

### Change of Location.

Dr. J. E. Jones of Dublin is now located at Boyce.

Dr. J. R. Hawkins of Houston has located at Port Lavaca.

Dr. J. W. Palmer of Windhem has moved to Uvalde, Texas.

Dr. D. Emroy Allen has moved from Newark to Polytechnic.

Dr. O. E. Looney of Illinois Bend, Texas, has located in Odell.

Dr. W. Q. Brown of Stratford, Texas, has located in Beeville.

Dr. Charles Stevenson of Kaufman will soon move to Houston.

Dr. Oliver and family of Farwell have decided to locate in Lorenzo.

Dr. S. D. Luse and family have moved to Poteet, Texas, from Sabinal.

Dr. Otis King and son of Alexander, Texas, have moved to La Pryor.

Dr. Grace and wife have left Port Lavaca for their future home in El Paso.

Dr. V. P. Randolph of Temple has located at Walburg to practice medicine.

Dr. Neathery, formerly of Haskell, is making a new home at Gainesville.

Dr. J. W. Blasdell of Lockhart has returned to Ballinger, where he will make his home.

Dr. J. H. Hendrix and family of Edgewood will make their future home in Bynum, Hill county.

Dr. Westbrook of Archer City will move to Mexia, where he will engage in the practice of medicine.

Dr. Blackwell of Beattie has moved to Sipe Springs. He will be in partnership with Dr. E. W. Duke.

Dr. Willis M. Moore of Hamilton has moved to Jonesboro, where he will engage in the practice of medicine.

Dr. S. S. Robinson, late of San Marcos, has decided to locate in Burnet for the purpose of practicing medicine.

Dr. and Mrs. J. H. Lander of Greenville have moved to Beeville, where Dr. Lander will be in partnership with Dr. R. M. Prather.

### Election of Officers in County Medical Societies.

The following officers were elected in various medical societies for the ensuing year:

The County Medical Society held its annual meeting in Kaufman Tuesday. The new officers-elect are: Dr. H. M. Phillips, president; Dr. W. F. Alexander, vice president; Dr. B. J. Hubbard, secretary-treasurer for the ninth consecutive term; Dr. U. P. Hackety, censor; Dr. B. J. Hubbard, delegate for two years to State Association, and Dr. W. J. Pollard, alternate delegate.

The McLennan County Medical Society. Officers for the ensuing year were elected as follows: President, H. T. Aynesworth, Waco; vice president, J. W. Gidney, West; secretary-treasurer, Doyle L. Eastland, Waco; delegate to the State Association meeting, Dr. J. M. Witt, Waco; board of censors, Carl Lovelace, I. E. Colgin and H. F. Connally.

New officers of the Tarrant County Medical Society of Fort Worth, Texas, elected to serve one year, are: Dr. J. A. Gracey, president; Dr. C. O. Harper, vice president; Dr. James J. Richardson, secretary, and Dr. W. R. Thompson, treasurer.

The Collin County Medical Society of McKinney, Texas: Dr. J. E. Gibson, president; Dr. D. F. Houston, vice president; Dr. E. L. Burton, secretary. Dr. Ben F. Largent was elected delegate to the State Medical Association, with Dr. J. C. Erwin as an alternate.

The Ellis County Medical Society of Waxahachie: Dr. J. L.

Rains of Bardwell, president; Dr. R. L. Hall of Italy, vice president; Dr. E. F. Gough of Waxahachie, secretary-treasurer; Dr. S. H. Watson of Waxahachie, censor; Dr. Z. T. Bunday of Milford, delegate to the State Association; Dr. J. S. Berry of Waxahachie, alternate delegate.

The Fannin County Medical Society: President, Dr. J. F. Rayburn; vice president, Dr. J. E. Norman of Trenton; secretary-treasurer, Dr. E. H. H. Foster; delegate, Dr. H. A. McDaniel; alternate, Dr. A. B. Kennedy; councillors, Dr. C. A. Gray and Dr. J. C. Carleton.

The Bexar County Medical Society of San Antonio, Texas: Dr. Conn L. Milburn, vice president; Dr. W. H. Hargis, secretary; Dr. L. B. Jackson, treasurer; Dr. Charles D. Dixon, delegate to State Medical Association; Dr. R. L. Dinwiddie, alternate; Dr. Frank Paschal, censor for the next three years. Dr. Dorbandt succeeds Dr. Charles D. Dixon as president.

The Hood-Somervell Medical Society of Granbury, Texas; H. L. Wilder of Glen Rose, president; T. H. Dabney of Granbury, vice president; E. C. Price of Tolar, secretary.

The Montague County Medical Society of Montague, Texas: Dr. D. W. Clark, president, Montague, Texas; Dr. L. E. Petty, vice president, Forestburg; Dr. C. F. Young, secretary-treasurer, Bowie; Dr. J. T. Lawson, Bowie, delegate to State convention; Dr. N. W. Crain, Nocona, alternate to State convention; censors, Dr. W. W. Davis, Nocona; Dr. E. E. Johnson, Mallard; Dr. H. F. Wilton, Nocona.

The Walker County Medical Society of Huntsville, Texas: President, Dr. M. E. Curtis; vice president, Dr. W. E. Fowler; secretary, Dr. J. W. Thomas; delegates, Drs. L. H. Bush and B. A. Autrey; censors, Drs. C. M. Tinsley, R. A. Autrey and J. P. Hendrick.

The Nueces County Medical Society of Corpus Christi, Texas: Dr. S. T. Dodge, president; Dr. L. Kaffie, vice president; Dr. A. W. Davisson, secretary-treasurer.

The Williamson County Medical Society: Dr. W. L. Helms of Jonah, president; Dr. W. G. Retters of Georgetown, secretary and treasurer. Dr. D. M. Cooke of Granger was elected censor and Dr. E. M. Wood of Georgetown, delegate to the State Medical Association.

Polk County Medical Society of Livingston, Texas: President, Dr. M. J. Taylor; vice president, Dr. Pullen; secretary-treasurer, Dr. T. H. Bates; censors, H. Bergman of Livingston, Dr. Stewart of Corrigan, and Dr. Maxwell of Shepherd. Dr. C. H. Robinson was chosen as the delegate to the State Medical Association which meets in Fort Worth.

The Franklin County Medical Association of Mt. Vernon, Texas: Dr. J. M. Fleming, president; Dr. Geo. Stephens, secretary; Drs. W. C. Crutcher and P. N. Davis, censors.

Johnson-Hill County Doctors' League: A. D. Ward, president, Blanton; J. M. Warren, secretary, Blum.

The Milam County Medical Society: Dr. E. E. Best of Cameron, president; Dr. W. R. Newton of Cameron, vice president; Dr. G. B. Taylor of Cameron, secretary and treasurer; Dr. C. J. Stanley of Buckholts, new censor; Dr. G. W. Mullins of Milano, delegate to the State convention; Dr. L. L. Lee of Thorndale, alternate delegate.

The Johnson County Medical Society of Cleburne, Texas: Dr. W. P. Ball, president; Dr. A. Y. Easterwood, first vice president; Dr. M. Dennis, second vice president; Dr. C. L. Edgar, secretary-treasurer; Dr. B. H. Turner, censor.

The Navarro County Medical Society: Dr. McClung of Corsicana, president; Dr. Carter of Powell, vice president. Dr. Cross of Corsicana was re-elected as secretary and treasurer, with Dr. Hanks of Corbet for censor for 1916, and Dr. Newton of Corsicana, censor for 1917. Dr. Shell of Corsicana was elected as a delegate, and Dr. Slater of Navarro as alternate to State Medical Society that meets in Fort Worth in May.

The DeWitt-Lavaca and Victoria-Calhoun Medical Societies: Dr. Marvin Duckworth, Cuero, president; Dr. J. E. Pridgen, Thomaston, vice president; Dr. B. J. Norwierski, Yorktown, secretary-treasurer; Dr. G. W. Allen, Yorktown, delegate to the State Medical Association to convene next May at Galveston; Dr. J. M. Lackey, Cuero, alternate; Dr. Lackey was also elected censor to fill the unexpired term of Dr. Finney.

The Delta County Medical Society: Dr. E. B. Wheat, president; Dr. T. M. Darwin, vice president; Dr. C. C. Taylor, secretary and treasurer; Dr. C. T. Bradford, censor for three years; Dr. L. D. Wood, delegate to State Medical Association; Dr. D. O. Lowry, alternate.

At the annual meeting of the Smith County Medical Society the following officers were elected for the ensuing year: President, Dr. G. G. Bell, Tyler; vice president, Dr. L. P. Tenney, Troup; board of censors, Drs. J. D. Phillips, B. F. Bell and H. H. Wisdom; delegate to State Association, Dr. L. P. Tenney.

The following officers were elected at the meeting of the Hidalgo County Medical Society at Pharr: President, Dr. Chas. B. Buck, Mercedes; vice president, Dr. J. R. Miller, Edenburg; secretary-treasurer, Dr. W. R. Dashiell, Mission.

